# Induction of Osteopontin by Dengue Virus-3 Infection in THP-1 Cells: Inhibition of the Synthesis by Brefelamide and Its Derivative

**DOI:** 10.3389/fmicb.2017.00521

**Published:** 2017-03-29

**Authors:** Dyshelly N. Pascapurnama, Hannah K. M. Labayo, Isolde Dapat, Divya D. Nagarajegowda, Jingge Zhao, Jing Zhang, Osamu Yamada, Haruhisa Kikuchi, Shinichi Egawa, Yoshiteru Oshima, Haorile Chagan-Yasutan, Toshio Hattori

**Affiliations:** ^1^Division of International Cooperation for Disaster Medicine, International Research Institute of Disaster Science, Tohoku UniversitySendai, Japan; ^2^Division of Emerging Infectious Diseases, Graduate School of Medicine, Tohoku UniversitySendai, Japan; ^3^Division of Disaster-related Infectious Diseases, International Research Institute of Disaster Science, Tohoku UniversitySendai, Japan; ^4^Research and Development Center, FUSO Pharmaceutical Industries, LtdOsaka, Japan; ^5^Laboratory of Natural Product Chemistry, Graduate School of Pharmaceutical Sciences, Tohoku UniversitySendai, Japan; ^6^Graduate School of Health Science and Social Welfare, Kibi International UniversityTakahashi, Japan

**Keywords:** dengue virus-3, THP-1 cell, 293T cell, osteopontin, brefelamide

## Abstract

Osteopontin (OPN) is a multifunctional matricellular protein produced by a broad range of cells including osteoclasts, macrophages, T cells, endothelial cells, and vascular smooth muscle cells. OPN modulates various physiological and pathological events such as inflammation, wound healing, and bone formation and remodeling. Dengue virus (DENV) infection causes an increase in plasma OPN levels, which is correlated with the severity of symptoms and coagulation abnormalities. DENV infection also induces *OPN* gene expression in human macrophages. This study investigated the inhibitory effects of brefelamide and its methyl ether derivative on DENV-3 by measuring changes in OPN levels in human THP-1 and 293T cell lines infected at different multiplicities of infection and post-infection time points. *OPN* mRNA expression and viral RNA were detected by reverse transcriptase quantitative real-time PCR, whereas protein level was determined by enzyme-linked immunosorbent assay. We found that viral copy number was higher in 293T than in THP-1 cells. However, THP-1 constitutively expressed higher levels of OPN mRNA and protein, which were enhanced by DENV-3 infection. Brefelamide and its derivative suppressed OPN production in DENV-3 infected THP-1 cells; the effective doses of these compounds had no effect on uninfected cells, indicating low cytotoxicity. These results suggest that brefelamide and its methyl ether derivative have therapeutic effects in preventing inflammation, coagulopathy, and fibrinolysis caused by OPN upregulation induced by DENV-3 infection.

## Introduction

Dengue is mosquito-borne, acute, febrile disease prevalent in tropical and subtropical areas. The number of reported dengue cases has been increasing annually; 3.97 billion people in 128 countries where dengue is endemic are at high risk (Brady et al., 2012; Bhatt et al., 2013). Each year, there are an estimated 390 million dengue cases worldwide and 250,000 fatal cases of dengue hemorrhagic fever and dengue shock syndrome (DHF/DSS). Dengue is caused by dengue virus (DENV), a single-stranded, positive-polarity enveloped RNA flavivirus (Henchal and Putnak, 1990). The disease itself is transmitted through the bite of the blood-feeding mosquito *Aedes aegypti*. There are four serotypes of DENV that cause illness (DENV-1 to -4), which presents a wide spectrum of clinical symptoms although the majority of cases are asymptomatic. Dengue fever is a flu-like syndrome, but severe DHF is characterized by coagulopathy and increased vascular fragility and permeability. The highest risk is associated with DHF that progresses to DSS, whereby hypovolemic shock can lead to death (Abel et al., 2012). Although the molecular mechanisms underlying the progression of dengue illness are not fully understood, they are presumed to be associated with increased coagulation and fibrinolytic activity during DENV infection (Avila-Aguero et al., 2004; Huerta-Zepeda et al., 2008) resulting from elevated levels of thrombin-antithrombin complex, D-dimer (fibrin degradation product), tissue plasminogen activator, and prothrombin fragment (Suharti et al., 2002; Wills et al., 2002).

Osteopontin (OPN) is a calcium-binding glycophosphoprotein that was originally isolated from bone and mediates bone remodeling and tissue debridement (Giachelli and Steitz, 2000). OPN has been implicated in pathological and physiological processes such as cell proliferation and endothelial cell migration (Senger et al., 1996; Chagan-Yasutan et al., 2011); in addition, it is expressed by macrophages and plays an important role in immunity, inflammation, tumor progression, and cell viability (Mazzali et al., 2002; El-Tanani et al., 2006) as well as cell adhesion, proliferation, invasion, and apoptosis in tissue fibrosis (Kato et al., 2014). We previously reported the upregulation of OPN in the plasma in DENV-infected patients, implying a role for OPN in disease progression (Chagan-Yasutan et al., 2014). OPN was also found to be overexpressed in DENV-infected human macrophages (Moreno-Altamirano et al., 2004). Full-length OPN is secreted into the extracellular matrix, where it modulates cell function by interacting with receptors, proteases, and hormones and matrix proteins such as collagen; it is also linked to activation of the coagulation pathway and fibrinolysis (Shinohara et al., 2008).

Phorbol 12-myristate 13-acetate (PMA) induces *OPN* gene expression in smooth muscle cells (Panda et al., 1997) and is frequently used as a control in inhibitor studies related to OPN expression. PMA induces hyperacetylation of histones H3 and H4 in the proximal region of the *OPN* promoter, which causes the binding of activator protein (AP)-1. PMA was also reported to enhance the recruitment of RNA pol II and TFIIB to the AP-1-binding region of the *OPN* promoter, resulting in upregulation of *OPN* expression (Sharma et al., 2010). 3-Hydroxy-3-methylglutaryl coenzyme A (HMG-CoA) reductase inhibitors (also known as statins) inhibit OPN protein synthesis in ovarian clear cell carcinoma cells *in vitro* and *in vivo* (Takemoto et al., 2001; Minoretti et al., 2006; Matsuura et al., 2010). Statins act as competitive inhibitors of endogenous HMG-CoA that deplete circulating isoprenoid levels, which can affect the expression of glycoproteins such as OPN (Matsuura et al., 2011). Statins also inhibit the small GTP-binding proteins Rho, Ras, and Rac whose membrane localization and function depend on isoprenylation (Liao, 2002). It has been suggested that statins bind to the *OPN* promoter and suppress gene expression (Matsuura et al., 2011).

Brefelamide and its methyl ether derivative have been reported to inhibit *OPN* expression (Zhang et al., 2016b) in DENV-infected cells. This was further investigated in the present study using two different human cell lines. We hypothesized that downregulation of OPN in DENV-infected cells would reduce cell invasiveness, coagulopathy, inflammation, and fibrinolysis, thereby preventing exacerbation of the illness.

## Materials and Methods

### Cell Lines and Culture

Human embryonic kidney 293T cells (DuBridge et al., 1987) and THP-1 monocytic cells derived from acute monocyte leukemia patients (Tsuchiya et al., 1980) were obtained from the American lType Culture Collection (Manassas, VA, USA). The former was maintained in high-glucose Dulbecco’s Modified Eagle’s medium containing L-glutamine and Phenol Red (Wako Pure Chemical Industries, Osaka, Japan) and the latter in Roswell Park Memorial Institute 1640 medium (Wako Pure Chemical Industries). Both media were supplemented with 10% of heat-inactivated fetal bovine serum (FBS) (Thermo Fisher Scientific, Waltham, MA, USA). Cells were cultured at 37°C in a humidified atmosphere of 5% CO_2_.

### DENV Infection

Dengue virus-3 was isolated from a patient with dengue fever at San Lazaro Hospital in Manila, Philippines as previously reported (Chagan-Yasutan et al., 2013). Viral titer was measured with the plaque assay using Vero cell lines. Viral concentration was determined as plaque-forming units per ml and was used to calculate the multiplicity of infection (MOI). Adherent 293T cells were seeded in a 12-well cell culture plate coated with BioCoat poly-D-lysine (Corning, Tokyo, Japan) at 2 × 10^5^ cells/well in 1 ml of growth medium supplemented with FBS, and cultured overnight at 37°C in a humidified atmosphere of 5% CO_2_. The following day, 0.5 ml of medium was removed from each well and DENV-3-containing medium was added for infection. DENV MOIs were adjusted to 0.01, 0.03, and 0.1. PMA (Wako Pure Chemical Industries, Osaka, Japan) with concentration of 100 ng/ml was used as a positive control to induce OPN expression. Cells were cultured at 37°C/5% CO_2_ for 1.5 h with rocking every 30 min to ensure infection. After 1.5 h, the viral suspension was removed and wells were washed twice with culture medium (Diamond et al., 2000). A 1-ml volume of new culture medium was added and cells were cultured until they were harvested on days 1, 2, and 3 post-infection.

THP-1 cells were seeded in a Nunc cell culture tube (Thermo Fisher Scientific) at 2 × 10^5^ cells/tube in 1 ml of growth medium supplemented with FBS. The next day, the tubes were centrifuged at 1200 rpm for 5 min and the spent medium was decanted; 0.1 ml of DENV-3 diluted in culture medium was added to each tube at final MOIs of 0.01, 0.03, and 0.1. PMA was used as a positive control to induce OPN expression. The tubes were incubated for 1.5 h with rocking every 30 min; the cells were washed twice with medium (Kurane et al., 1990) and cultured in 1 ml of fresh medium before they were harvested on days 1, 2, and 3 post-infection.

### Collection of DENV-Infected Cells and RNA Extraction

The supernatant from cell cultures was transferred to 1.5-ml tubes (Eppendorf, Hamburg, Germany) and stored at -80°C. The 293T cells were trypsinized using 0.25% trypsin-EDTA (Thermo Fisher Scientific), transferred to 1.5-ml tubes, and washed with phosphate-buffered saline. Meanwhile, THP-1 cells were collected by centrifugation. Cell viability was determined based on exclusion of Trypan Blue (Bio-Rad, Hercules, CA, USA). The cells were centrifuged at 1200 rpm for 5 min and RNA was extracted by adding 200 μl of homogenization solution from the Maxwell 16 LEV simplyRNA Cells kit and Tissue kit (#AS1270 and #AS1280, respectively; Promega, Madison, WI, USA). Samples were stored at -80°C.

### Determination of DENV Copy Number by Real-Time Quantitative (RT-q)PCR

Viral copy number in culture supernatants and cell lysates was measured by RT-qPCR as previously described (Chagan-Yasutan et al., 2013) using the RNA UltraSense One-Step Quantitative RT-PCR System (Invitrogen, Carlsbad, CA, USA) and Thermal Cycler Dice Real Time System (Takara Bio, Otsu, Japan) according to the manufacturers’ protocols. Primers and hydrolysis probes specific to the 3′ untranslated region of each of the four DENV genotypes have been previously published (Chagan-Yasutan et al., 2013). The forward and reverse primers were as follows 5′-AAGGACTAGAGGTTAGAGGAGACCC-3′ and 5′-CGTTCTGTGCCTGGAATGATG-3′ (Warrilow et al., 2002). The TaqMan probe was labeled at the 5′ and 3′ ends with a 6-carboxyfluorescein (FAM) reporter and Black Hole quencher (BHQ)-1 (i.e., 5′[FAM]–TGGGARAGACCAGAGATCCTGCTGTCT–[BHQ1]3′). The copy numbers obtained by DENV RT-qPCR were linearly associated with plaque numbers (data not shown).

### Quantification of OPN mRNA Expression by RT-qPCR

Glyceraldehyde 3-phosphate dehydrogenase (GAPDH) was used as reference gene for normalization of expression levels and was amplified with the following forward and reverse primers: GAPDH-F, 5′-GCACCGTCAAGGCTGAGAAC-3′ and GAPDH-R, 5′-TGGTGAAGACGCCAGTGGA-3′. The Taqman probe was as follows: 5′[FAM]–TCCACGACGTACTCAGCGCCAGCAT–[BHQ1]3′. A forward and reverse primer set (SPP1-F, 5′-GATGAATCTGATGAACTGGTCACTG-3′ and SPP1-R, 5′-GGTGATGTCCTCGTCTGTAGCA-3′) and a probe (SPP1-P, 5′-[FAM]–CCACGGACCTGCCAGCAACC GAAGT–[BHQ1]3′) (Takara Bio) were used to quantify *OPN* mRNA (Qin et al., 2015). The mean fold change in *OPN* mRNA level was calculated as fold difference = 2^-ΔΔCt^.

### Determination of OPN Concentration by Enzyme-Linked Immunosorbent Assay (ELISA)

Osteopontin levels in the cell culture supernatant were quantified using an ELISA kit (R&D Systems, Minneapolis, MN, USA) according to the manufacturer’s instructions and as previously reported (Chagan-Yasutan et al., 2014). The supernatant was diluted eightfold for THP-1 cells and two–fourfold for 293T cells, and the final value was calculated as 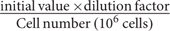 and was expressed as pg/ml/10^6^ cells.

### Cytotoxic Effects of Compounds A and B

Brefelamide, henceforth referred to as compound A (Kikuchi et al., 2005), is an aromatic amide isolated from methanol extracts of *Dictyostelium brefeldianum* and *D. giganteum* slime mold fruiting bodies; its derivative, henceforth referred to as compound B (Kikuchi et al., 2005; Zhang et al., 2016b), contains a methyl ether group (-OCH_3_). Both compounds were dissolved in dimethyl sulfoxide (DMSO) at a concentration of 50 mM and stored at -20°C. Aliquots of the stock solutions were further diluted with appropriate growth medium to the desired concentrations before use. The cytotoxic effect of the compounds is expressed as the 50% inhibitory concentration (IC_50_), which was determined with the CellTiter 96 Aqueous Non-Radioactive Cell Proliferation System (Promega). THP-1 and 293T cells were seeded in 96-well tissue culture plates at a density of 1.8 × 10^4^/well in 90 μl of the appropriate growth medium and incubated for 24 h at 37°C in a humidified 5% CO_2_ atmosphere. The following day, 10 μl of compounds A and B at concentrations ranging from 0.0001 to 10 mM were added to each well. After incubation for 24 h, 20 μl per well of 3-(4,5-dimethyl-2-yl)-5-(3-carboxymethoxyphenyl)-2-(4-sulfophenyl)-2H-tetrazolium salt/phenazine methosulfate solution was added to each well, followed by incubation for 4 h. The absorbance at 490 nm was recorded with an ELISA plate reader and the IC_50_ value was determined by identifying the *X*-axis value corresponding to one-half of the difference between the maximum and minimum absorbance values using GraphPad Prism v.6 software (GraphPad Inc., San Diego, CA, USA).

### Changes in OPN Level in Response to Compounds A and B in Uninfected Cells

Changes in OPN expression in uninfected THP-1 and 293T cell lines in response to treatment with compounds A and B were assessed. Each well in a 12-well tissue culture plate was seeded with 1.8 × 10^5^ cells. After overnight incubation, the compounds were added at final concentrations of 3–100 μM. Cells were harvested 3 days later. OPN mRNA and protein levels were quantified by RT-qPCR and ELISA, respectively, to determine the optimal concentration.

### Effects of Compounds A and B on DENV-Infected Cells

THP-1 and 293T cells were seeded in a Nunc cell culture tube and a 12-well cell culture plate, respectively, at 1.8 × 10^5^/well. After overnight incubation, cells were washed and incubated with fresh medium containing DENV-3 at an MOI of 0.1 or incubated without virus for 1.5 h. The inoculum was then removed and cells were washed twice with fresh medium, and compounds A and B were added at final concentrations of 3, 10, and 30 μM. As negative and positive controls, 0.2% DMSO and 0.3–1 μM statin (simvastatin, cat. no. S6196; Sigma-Aldrich, St. Louis, MO, USA), respectively, were added to the cells. On day 3 post-infection, culture supernatants and cell lysates were collected. Cell viability was evaluated by Trypan Blue exclusion and DENV copy number was determined by RT-qPCR. OPN mRNA and protein levels were quantified by RT-qPCR and ELISA, respectively.

### Statistical Analysis

A two-tailed *t*-test was used to compare group means, and *P* < 0.05 was considered statistically significant. All statistical analyses were performed using GraphPad Prism software.

## Results

### DENV Infection Induces OPN mRNA Expression

**FIGURE 1:**
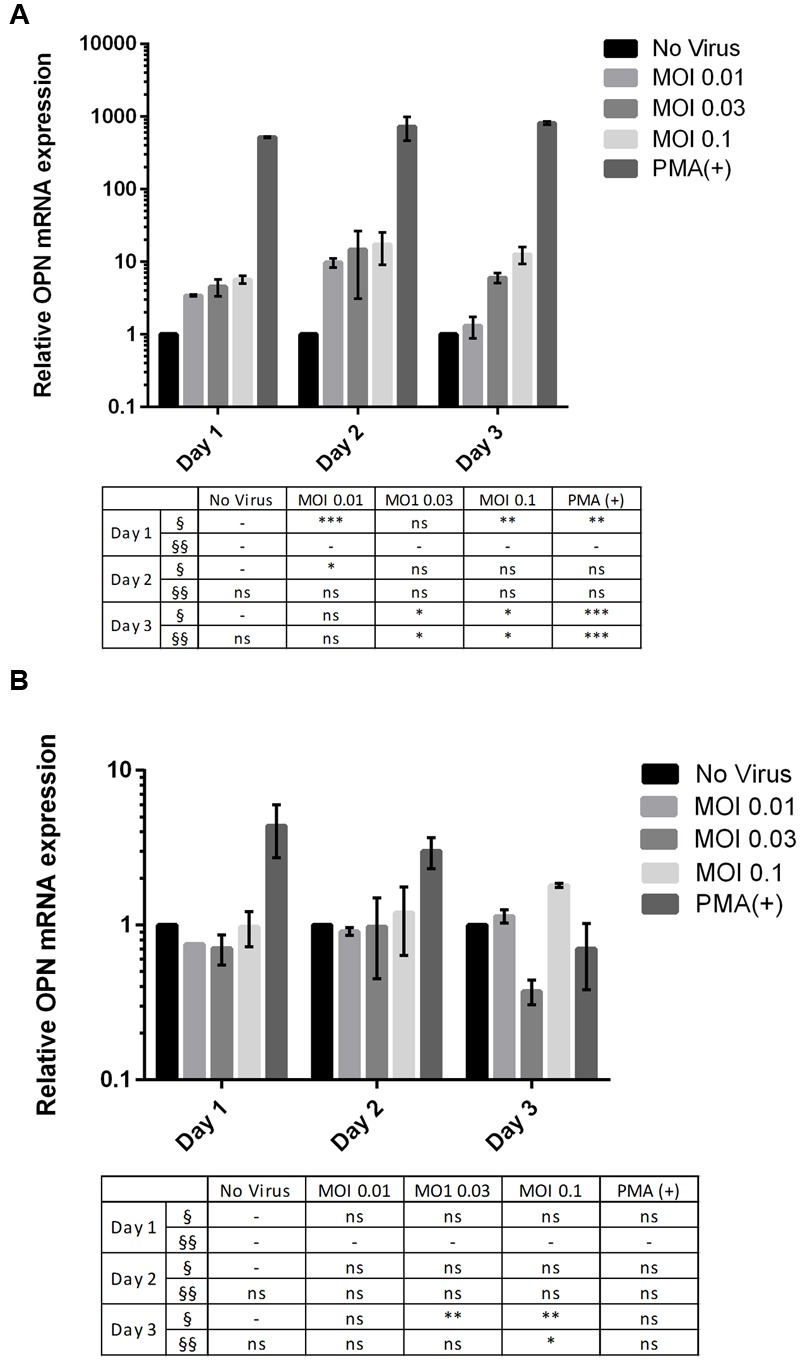
**Multiplicity of infection (MOI)- and time-dependent change in *OPN* mRNA expression upon dengue virus (DENV) infection. (A)** THP-1 and **(B)** 293T cells were infected with DENV-3 at various MOIs (range: 0.01–0.1). Phorbol 12-myristate 13-acetate (PMA) was used as a positive control. Cells were harvested daily (1–3 days). Total RNA was prepared from cell lysates and OPN levels were determined by RT-qPCR. GAPDH was used as reference gene to normalize the expression level. The table summarizes statistical analysis, ^∗^*P* < 0.05, ^∗∗^*P* < 0.01, ^∗∗∗^*P* < 0.001 vs. control (uninfected cells; unpaired two-tailed *t*-test). Data represent mean ± SEM. § and §§ represent MOI- and time-dependent variables, respectively.

*OPN* mRNA expression increased in DENV-infected THP-1 cells (**Figure 1A**) in a MOI-dependent manner, as determined by RT-qPCR. Cells infected at MOI of 0.1 resulted 12.59-fold higher OPN levels after 3 days than those infected at MOI = 0.01 (*P* < 0.005). These results indicate that DENV infection induces OPN synthesis in THP-1 cells. A similar trend was observed in 293T cells, although the difference in OPN expression level between cells infected at an MOI of 0.1 and uninfected cells was smaller (1.8-fold; *P* < 0.005) (**Figure 1B**). Thus, an MOI of 0.1 for 3 days was used for subsequent experiments in both THP-1 and 293T cells.

### DENV Infection Induces OPN Synthesis

**FIGURE 2:**
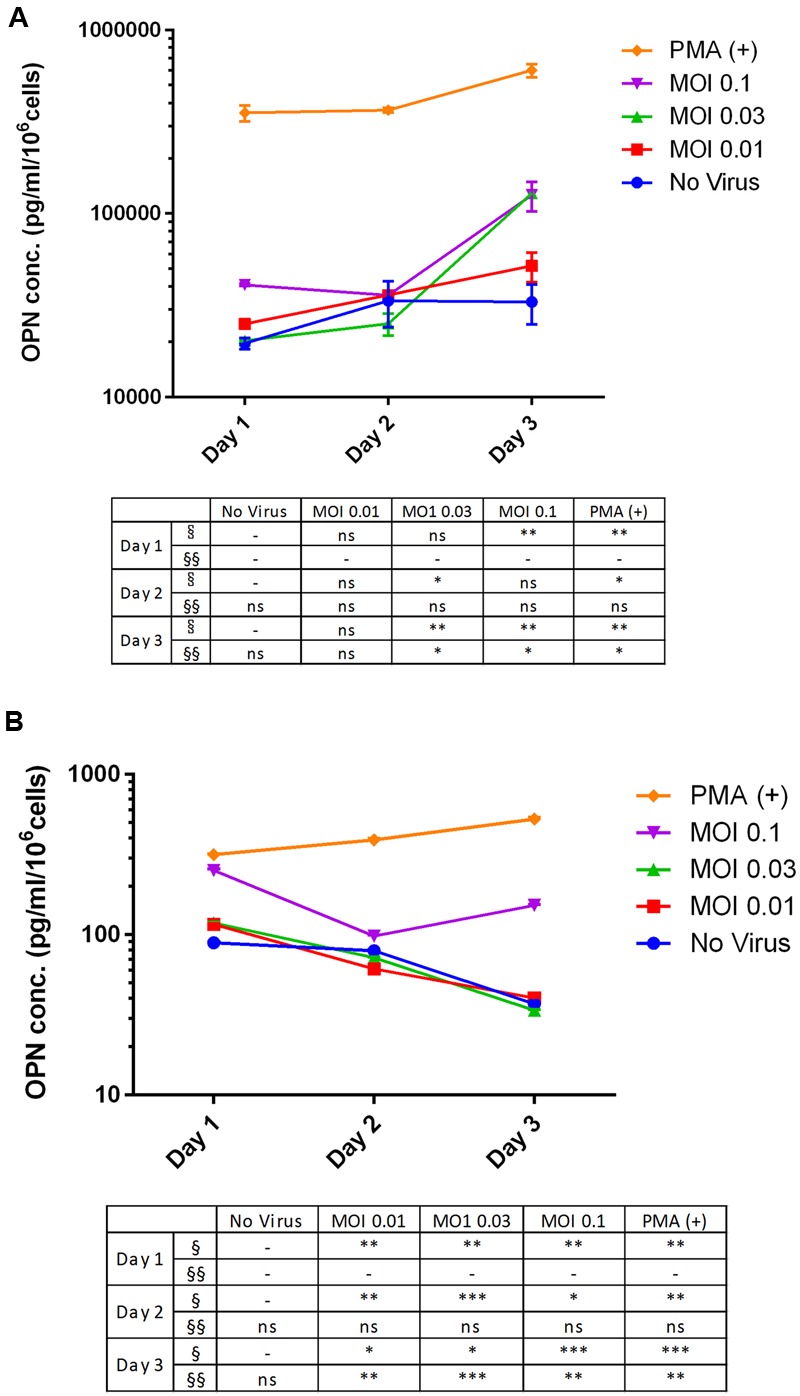
**Multiplicity of infection- and time-dependent change in OPN protein expression upon DENV infection. (A)** THP-1 and **(B)** 293T cells were infected with DENV-3 at various MOIs (range: 0.01–0.1). PMA was used as a positive control. Cells were harvested daily (1–3 days) and OPN protein levels in the supernatant were measured by ELISA. Viability was simultaneously evaluated to normalize the results. Normalized OPN levels in each sample are expressed as pg/ml/10^6^ cells. The table summarizes statistical analysis, ^∗^*P* < 0.05, ^∗∗^*P* < 0.01, ^∗∗∗^*P* < 0.001 vs. control (uninfected cells; unpaired two-tailed *t*-test). Data represent mean ± SEM. § and §§ represent MOI- and time-dependent variables, respectively.

We investigated changes in OPN expression in response to DENV infection at different MOIs and infection times using THP-1 and 293T cell lines. OPN level in the THP-1 cell culture supernatant increased upon DENV infection (**Figure 2A**). The results at day 3 using MOI at 0.03 and 0.1 are significantly higher than other cultures (3.9-fold at MOI 0.03 and 3.83-fold at MOI 0.1; *P* < 0.001 compared to no virus group). The significant difference was also observed at day 1 at MOI 0.1. In DENV-infected 293T cells (**Figure 2B**), cultures with higher MOIs have higher OPN levels when compared to controls on day 1 post-infection (2.8-fold at MOI 0.1; *P* < 0.001). However, in contrast to THP-1 cells, OPN levels in the supernatant of 293T cells decreased gradually as the incubation time post-infection increased.

**FIGURE 3:**
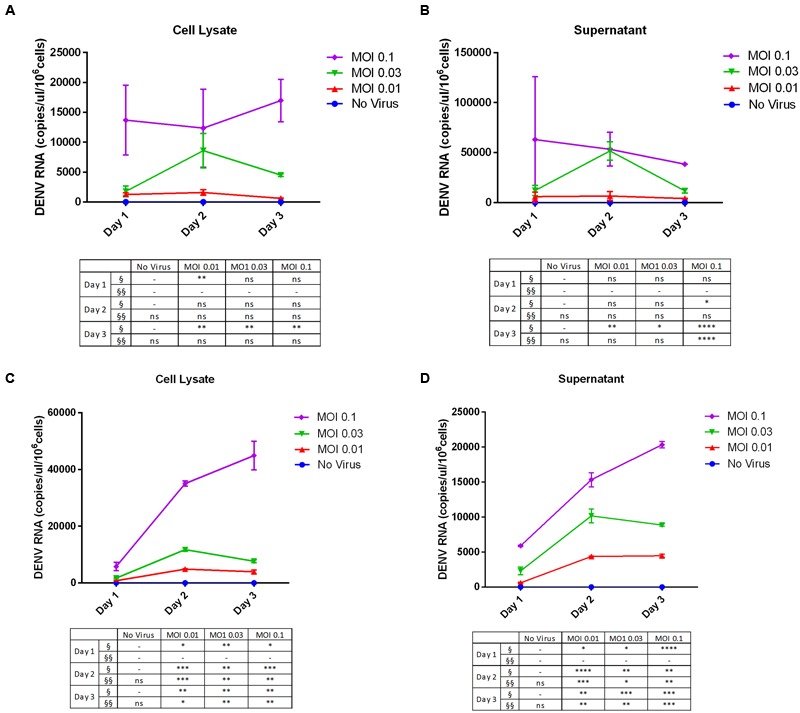
**Dengue virus RNA copy number in infected cell lines. (A,B)** THP-1 and **(C,D)** 293T cells were left uninfected or infected with DENV-3 at various MOIs (range: 0.01–0.1). Cells were harvested at various post-infection time points (days 1, 2, and 3). Total RNA was extracted from cell lysates and the culture supernatant and DENV genome copy number was determined by RT-qPCR. The table summarizes statistical analysis, ^∗^*P* < 0.05, ^∗∗^*P* < 0.01, ^∗∗∗^*P* < 0.001, ^∗∗∗∗^*P* < 0.0001 vs. control (uninfected cells; unpaired two-tailed *t*-test). Data represent mean ± SEM. § and §§ represent MOI- and time-dependent variables, respectively.

In addition, we determined the viral copy number of DENV RNA in supernatants and cell lysates by RT-qPCR. For THP-1 cells, fewer copies of DENV RNA were detected in the cell lysate (**Figure 3A**) than in the supernatant (**Figure 3B**). The rate of viral replication was high on day 1 at an MOI of 0.1, and we did not detect any changes in copy number over time. However, in both lysates and supernatant samples, DENV replication (MOI = 0.1, day 3) was increased relative to lysates and the culture supernatant of uninfected cells. On the other hand, there were more copies of DENV RNA in 293T cell lysates (**Figure 3C**) than in the culture supernatant (**Figure 3D**), especially at an MOI of 0.1 on day 3. Both cell lysates and culture supernatants showed MOI-dependent increases in DENV RNA copy number by RT-qPCR.

### Compounds A and B Are Less Cytotoxic to THP-1 and 293T Cells than Statins

The cytotoxicity of compounds A and B relative to a statin (simvastatin) was determined based on their IC_50_ values. The IC_50_ of compound A was 102.1 μM in THP-1 cells and 32.2 μM in 293T cells; for compound B, the values were 27.8 and 9.9 μM, respectively; and for simvastatin the values were 14.1 and 9.8 μM, respectively. These results indicate that compounds A and B are less cytotoxic to these cell lines than a clinically available statin.

### Compounds A and B Inhibit OPN mRNA Expression

**FIGURE 4:**
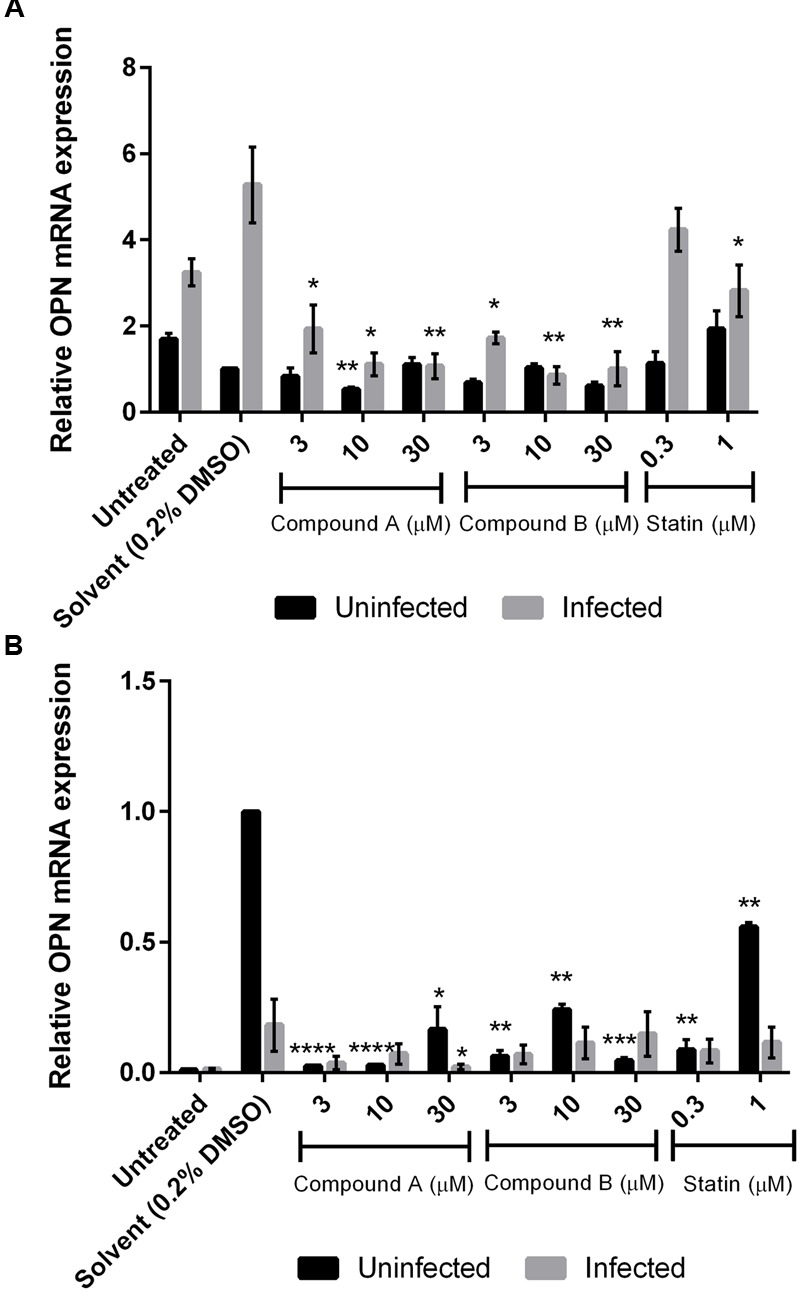
**Changes in *OPN* mRNA expression by treatment with compounds A and B in DENV-infected cells.** Uninfected and DENV-infected THP-1 **(A)** or 293T **(B)** cells were left untreated or treated with compound A or compound B (range: 3–30 μM), or statin (range: 0.3–1 μM) for 72 h. Total RNA was extracted from cell lysates and analyzed by RT-qPCR. GAPDH was used as reference gene to normalize expression levels. Results show the mean of two independent experiments. ^∗^*P* < 0.05, ^∗∗^*P* < 0.01, ^∗∗∗^*P* < 0.001, ^∗∗∗∗^*P* < 0.0001 vs. control (0.2% DMSO; unpaired two-tailed *t*-test). Data represent mean ± SEM.

Compound A inhibited *OPN* mRNA expression in a dose-dependent manner in DENV-infected THP-1 cells but had no effect on uninfected control cells. Similar results were obtained for compound B (*P* < 0.05) (**Figure 4A**). In contrast, both compounds reduced *OPN* mRNA levels in uninfected but not in DENV-infected 293T cells (**Figure 4B**). The addition of the solvent DMSO also stimulated *OPN* mRNA expression in both cell lines, which was especially evident in 293T cells; however, this effect was abrogated in the presence of compounds A and B.

### Compounds A and B Inhibit OPN Production in Uninfected and DENV-Infected 293T and THP-1 Cells

**FIGURE 5:**
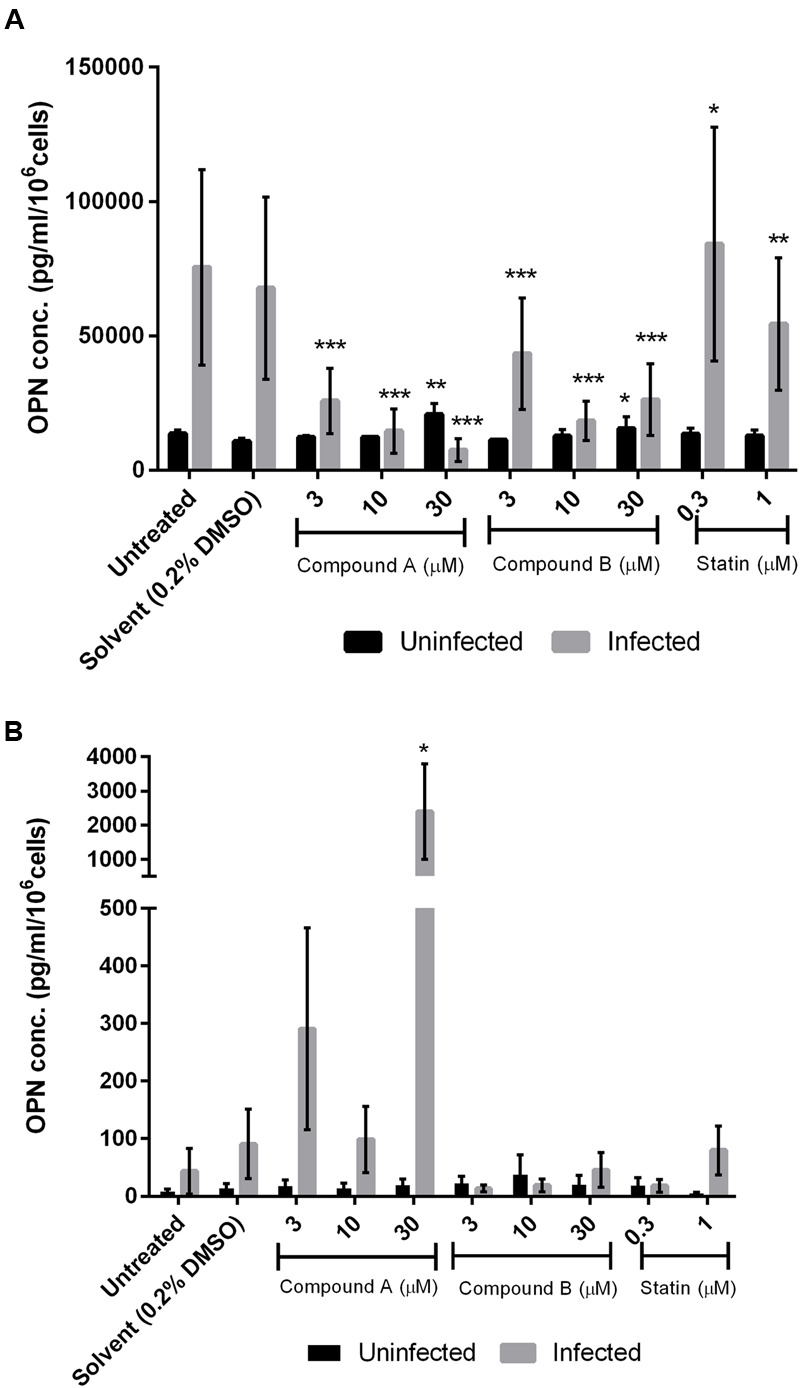
**Effect of compounds A and B on OPN protein level in DENV-infected cells.** Uninfected and DENV-infected THP-1 **(A)** or 293T **(B)** cells were left untreated or treated with compound A or compound B (range: 3–30 μM), or statin (range: 0.3–1 μM) for 72 h. Cells were then harvested and OPN levels in the culture supernatant were determined by ELISA. Viability was simultaneously measured to normalize the results. Normalized OPN levels in each sample are expressed as pg/ml/10^6^ cells. Results show the mean of two independent experiments. ^∗^*P* < 0.05, ^∗∗^*P* < 0.01, ^∗∗∗^*P* < 0.001 vs. control (0.2% DMSO; unpaired two-tailed *t-*test). Data represent mean ± SEM.

We evaluated OPN levels in the supernatant of uninfected and DENV-infected THP-1 and 293T cell cultures treated for 3 days with various concentrations of compounds A and B and simvastatin by ELISA. Treatment with compound A had no effect on OPN levels in uninfected THP-1 cells but it induced a dose-dependent reduction in cells infected with DENV (**Figure 5A**); 10 μM compound A reduced OPN levels to a value comparable to those observed in cultures of uninfected DMSO-treated cells (from 67848.9 to 12247.9 pg/ml/10^6^ cells; *P* < 0.0005). Similarly, OPN levels in uninfected cells were unaffected by compound B but were markedly downregulated in DENV-infected cells (**Figure 5A**). The greatest decrease in OPN expression (from 67848.9 to 18267.8 pg/ml/10^6^ cells, *P* < 0.0005) was observed by treatment with 10 μM compound B. In fact, OPN level was upregulated in uninfected cells by treatment with 30 μM compound B, suggesting a cytotoxic effect (i.e., the IC_50_ of compound B in THP-1 cells is 9.9 μM). Simvastatin had no effect on OPN level in DENV-infected THP-1 cells.

Compound A did not inhibit OPN expression in uninfected or DENV-infected 293T cells; adding 30 μM of compounds to the uninfected cells stimulated OPN expression, showing cytotoxicity effect (**Figure 5B**). In contrast, compound B (3 μM) reversed the upregulation of OPN expression induced by DENV infection in 293T cells (from 90.8 to 13.4 pg/ml/10^6^ cells), although the significance of this decrease was not statistically proven (**Figure 5B**). Treatment with simvastatin (0.3 μM) also suppressed OPN production in DENV-infected 293T cells.

### Compound B Inhibits DENV Replication

**FIGURE 6:**
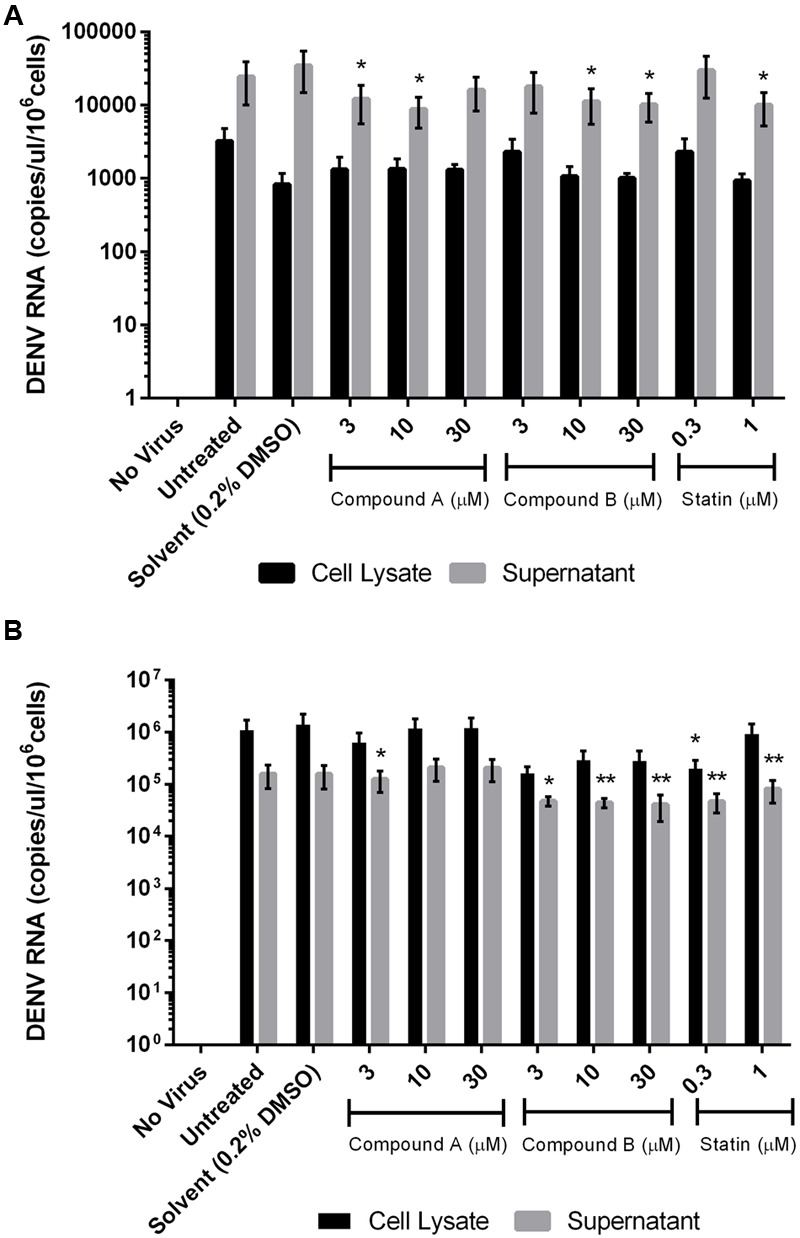
**Effect of compounds A and B on DENV RNA copy number in infected cells.** Uninfected and DENV-infected THP-1 **(A)** or 293T **(B)** cells were left untreated or treated with compound A or compound B (range: 3–30 μM), or statin (range: 0.3–1 μM) for 72 h. Total RNA was extracted from cell lysates and DENV RNA was detected by RT-qPCR. Results show the mean of two independent experiments. ^∗^*P* < 0.05, ^∗∗^*P* < 0.01 vs. control (0.2% DMSO; unpaired two-tailed *t*-test). Data represent mean ± SEM.

More copies of DENV RNA were detected in the culture supernatant as compared to the lysates of THP-1 cells. Compounds A and B (10 μM) suppressed DENV RNA levels in the THP-1 cell culture supernatant, but not lysates, by 75% relative to the control (**Figure 6A**). Simvastatin had no effect on virus replication as shown by the high copy number of DENV RNA in the culture supernatant of THP-1 cells. In contrast, more copies of DENV RNA were detected in the lysates than in the culture supernatant of 293T cells; and compound B, but not compound A, reduced DENV RNA copy number in the supernatant. Simvastatin (0.3 μM) inhibited viral replication in both the culture supernatant and in lysates of 293T cells (**Figure 6B**).

## Discussion

The results presented here show for the first time that DENV infection induces OPN production in THP-1 and 293T cells. The two cell lines responded differently to DENV infection. Infected THP-1 cells increased OPN production. On the other hand, viral replication was efficient in 293T cells but resulted in the release of less OPN into the supernatant. We also demonstrated that upregulation of OPN expression in response to DENV infection was suppressed by treatment with compounds A and B at non-cytotoxic concentrations.

Infection by any of the four DENV serotypes can cause asymptomatic infection, mild fever, or fatal DHF and DSS. The severity of infections may depend on soluble immune response mediators (Moreno-Altamirano et al., 2004). Chemokines and cytokines and their receptors as well as adhesion molecules—including interleukin (IL)-8; IL-1β; C-X-C motif chemokine ligand-1, -2, and -3; and C-C motif chemokine ligand-1—have been implicated in DENV pathogenesis and are upregulated upon DENV infection (Halstead, 1989). Moreover, serum IL-8 levels are correlated with disease severity (Bethell et al., 1998; Raghupathy et al., 1998; Juffrie et al., 2000). DENV infection causes damage to a variety of cell types such as macrophages, endothelial cells, and fibroblasts (Halstead, 1989; Kurane et al., 1992; Jessie et al., 2004) that normally produce OPN and can increase the levels that are released, thereby exacerbating inflammation and activating the coagulation pathway (Huerta-Zepeda et al., 2008).

OPN expression is associated with hematocrit levels and platelet counts, and reflects plasma leakage and thrombocytopenia in the critical phase of DENV infection. Thus, OPN level is a biomarker for tracking the progression of inflammation and coagulopathy during infection (Chagan-Yasutan et al., 2014). In the immune response to tuberculosis infection, OPN level is negatively correlated with lymphocyte count and memory T cell activation whose migration to tuberculosis lesions is induced by OPN-mediated signaling (Shiratori et al., 2016). Severe forms of DENV infection—especially secondary infection—are characterized by excessive inflammation. OPN is involved in inflammation, coagulopathy, and fibrinolysis (Chagan-Yasutan et al., 2014); preventing excessive inflammation by inhibiting OPN may thus block the progression of DENV infection.

THP-1 is a human monocytic cell line derived from an acute monocytic leukemia patient. These cells resemble and can differentiate into macrophages, which are the primary target of DENV (Halstead, 1989) along with endothelial, mast, and dendritic cells (Avirutnan et al., 1998; Ho et al., 2001; King et al., 2002). We found here that THP-1 but not 293T cells constitutively express OPN, which is consistent with the observation that OPN is produced by macrophages (Oyama et al., 2000). OPN mRNA is upregulated in monocyte-derived macrophages from healthy human donors 6 h after DENV-2 infection, and remains elevated until 72 h after infection (Moreno-Altamirano et al., 2004). In our study, DENV infection increased OPN mRNA and protein levels in both THP-1 and 293T cells, although with a more pronounced effect on the former. In 293T cells, significant changes in the level of OPN protein were observed at 24 h post-infection then levels started to decline the day after. The reasons for the decline are not clear. One possible explanation is that 293T cells normally do not produce OPN; thus, OPN expression and production may only be temporary and start to be downregulated thereafter. Further results showed that in 293T cells, the level of OPN protein is increased by DENV infection at higher MOIs, although OPN mRNA level is unchanged. This discrepancy is suggestive of an enhanced OPN translational efficiency in the absence of increased OPN promoter activity. Higher MOIs and longer incubation times resulted in higher OPN expression, specifically in THP-1 cells, suggesting that OPN is involved in the cellular response to DENV infection. Indeed, plasma OPN levels are associated with the severity of inflammation, coagulopathy, and exacerbation of DENV infection (Chagan-Yasutan et al., 2014).

293T cells showed higher replication of DENV in the cell lysate and culture supernatant than THP-1 cells, which may be explained by the large T antigen used for cell transformation. The 293T cell line derived from human embryonic kidney is highly transfectable (DuBridge et al., 1987). While kidney cells are not considered as primary target of DENV infection, the illness is associated with several kidney disorders such as renal failure, proteinuria, and hematuria (Lizarraga and Nayer, 2014). Thus, studying DENV infection using both THP-1 and 293T cells can provide insight into different aspects of the illness. The capability of THP-1 and 293T (including its parental HEK293) cells to be used for DENV infection experiments had been well described (Diamond et al., 2000; Bosch et al., 2002). Antibody-dependent enhancement (ADE) experiments in THP-1 can be conducted in the future to facilitate virus entry into host cells and increase the infectivity of the virus toward the cell by stimulating Fc receptors (Halstead, 1989). Very recently, non-ADE DENV infection of THP-1 cells was found to dysregulate extracellular matrix (Afroz et al., 2016). ADE is mediated by Fc receptors and the stimulation of the receptor alone may stimulate OPN synthesis; OPN production in DENV infection with and without antibody could be compared at higher MOIs.

Inhibiting OPN expression has been suggested as a promising therapeutic strategy against malignancies (Zhang et al., 2016b). Transcriptional regulation of *OPN* is complex and involves many factors, including AP-1, Ets, Myc, and v-Src (Hijiya et al., 1994; Wang et al., 2000). OPN is also downregulated upon activation of transforming growth factor (TGF)-β signaling, which is linked to cytostatic mechanisms (Zhang et al., 2016a). We found here that compounds A and B markedly reduced OPN mRNA and protein levels in THP-1 cells, although viral replication was not significantly affected at non-cytotoxic concentrations of compound A. Nonetheless, our results suggest that these compounds can be used to target OPN and suppress inflammation following DENV infection to prevent its progression to DHF and DSS.

Aberrant OPN expression has been linked to activation of the extracellular signal-regulated kinase (ERK) pathway, indicating that OPN is a downstream target of ERK/AP-1 signaling (Kim et al., 2003). Cellular stress has also been shown to induce the upregulation of OPN via ERK activation (Kato et al., 2014). Compound A suppressed the proliferation of astrocytoma cells by inhibiting ERK phosphorylation and epidermal growth factor-dependent activation of ERK signaling (Kikuchi et al., 2005; Honma et al., 2009). Similarly, compounds A and B may suppress OPN expression by inhibiting ERK phosphorylation, which could then block proliferation of DENV-infected cells.

To address the concern of potential side effects, we investigated the cytotoxicity of compounds A and B. High concentrations of both compounds (>30 μM) were toxic to DENV-infected THP-1 and 293T cells (>30 μM), an effect that was dose-dependent, reflecting their original purpose as an anticancer drug that induces cell growth arrest (Zhang et al., 2016b). Between the two compounds, A showed greater potential than B since the concentration at which it was most effective was much lower than its IC_50_ value. Independent pre-clinical investigations are required to further assess the toxicology of compounds A and B. It should be noted that both compounds showed modest suppressive effects on viral replication in THP-1 cells, while only compound B exerted this effect in 293T cells. In both cells, the compounds showed suppressive effect resulting in decreased viral copies in the supernatant, although viral copies in cell lysate were unchanged. The reasons were not clear but the compounds may inhibit the release of viral progeny by the infected cells. However, the relationship between viral replication and OPN suppression requires more detailed analysis in future studies. In addition, the anti-inflammatory, immunomodulatory, and antiviral properties of compounds A and B must be evaluated before they can be used in pre-clinical trials.

In summary, this study demonstrated that DENV infection induced OPN production in THP-1 and 293T cells. Treatment with compounds A and B abrogated this effect at non-cytotoxic concentrations. Further investigation is warranted to determine whether controlling OPN levels in patients with DENV infection by treatment with these compounds can prevent exacerbation of the illness to DHF and DSS.

## Author Contributions

DP performed the experiments, analyzed the data, and drafted the manuscript. HL and DN performed the DENV infection experiment and collected DENV stock. ID and JZ (fifth author) contributed to data analysis. JZ (sixth author), OY, and SE contributed to data interpretation regarding OPN inhibition by the compounds. HK and YO supplied the compounds. HC-Y and TH conceived and designed the study, edited the manuscript, and provided reagents.

## Conflict of Interest Statement

The authors declare that the research was conducted in the absence of any commercial or financial relationships that could be construed as a potential conflict of interest.
